# The Use of Self-Inflating Hygroscopic Tissue Expanders to Facilitate Osteosarcoma Removal in a Massasauga Rattlesnake (*Sistrurus catenatus*)

**DOI:** 10.1155/2020/8813911

**Published:** 2020-07-28

**Authors:** Kate E. Archibald, Brigid Troan, Dustin Smith, Larry J. Minter

**Affiliations:** ^1^Department of Clinical Sciences, North Carolina State University College of Veterinary Medicine, 1060 William Moore Drive, Raleigh, NC 27606, USA; ^2^Hanes Veterinary Medical Center, North Carolina Zoological Park, 4401 Zoo Parkway, Asheboro, North Carolina 27205, USA; ^3^Environmental Medicine Consortium, North Carolina State University College of Veterinary Medicine, 1060 William Moore Drive, Raleigh, North Carolina 27607, USA

## Abstract

A 0.34 kg adult female Massasauga rattlesnake (*Sistrurus catenatus*) was presented for evaluation of a subcutaneous mass affecting the ventral scales. The mass was diagnosed as a sarcoma via punch biopsy with no evidence of metastasis on diagnostic imaging. Surgical margins of 1-2 cm were planned to achieve complete excision of the neoplasm. A technique for tissue expansion was employed due to concerns regarding the ability to close the surgical site without excess tension or dehiscence. Two 27 mm diameter × 5 mm hygroscopic self-inflating tissue expanders were placed subcutaneously under the lateral scales adjacent to the mass. Maximum skin expansion occurred over a four-week period, and no direct negative effects were noted. Excision of the primary mass was performed routinely five weeks after implant placement. Primary closure of the defect was achieved with minimal tension by incorporating the expanded skin. While the surgery was successful with no evidence of metastasis, the snake died of sepsis two weeks postoperatively. This is the first report of the use of self-inflating hygroscopic tissue expanders to help close a surgical defect in a reptile.

## 1. Introduction

Reports of skin stretching and tissue expansion first appeared in the veterinary medical literature during the 1980s. The initial publications described the clinical utility of these methods in dogs and horses [1–3], methods which have since been refined for use in a variety of domestic species and applications [4–12]. Adapted from human reconstructive medicine, skin stretching and tissue expansion are two different methods that enable veterinary surgeons to close large dermal wounds or surgical defects in regions of high tension. Achieving primary closure prevents complications and associated wound management that is necessitated by secondary intention healing [13]. In domestic mammals, these techniques are most commonly applied to cutaneous defects of the distal limbs or in regions of significant skin loss on the axilla [8, 11, 12, 14, 15]. They are employed as part of a delayed reconstruction plan and can be used to augment or replace other tension-relieving surgical techniques such as orienting incisions parallel to tension lines, undermining, placing walking sutures, performing tension-releasing incisions, and using skin flaps or grafts. Unlike the immediate redistribution of load via tension-relieving sutures or releasing incisions, skin stretching relies on “mechanical creep,” the tissue's response to constant tension applied across the skin surface resulting in realignment of dermal collagen fibers and subsequent loss of extensibility [16]. Stretch is typically achieved through placement of an external device on the surface of the skin that applies controlled traction across a defect [15, 17–20]. In contrast to skin stretching, tissue expansion (also referred to as skin expansion) involves the placement of subcutaneous implants which expand tissue via “biological creep,” the mechanism by which constant loading gradually stimulates the formation of new dermal and epidermal elements [16]. Reportedly, proliferation occurs through both cellular and molecular mechanisms resulting in increased skin surface area over time. Compared to skin stretching, the benefits of tissue expansion include the production of new skin, better tissue compliance, and improved blood flow via angiogenesis [21].

Tissue expansion in both human and veterinary patients was historically performed via subcutaneous placement of a silicone balloon that was periodically expanded by the infusion of air or saline [22, 23]. This technique has met with variable success and popularity in veterinary patients due to the cost, relative size of available implants, and necessity of maintaining a SQ or external port for intermittent expansion [4, 15, 24, 25]. However, the development of small hygroscopic self-inflating tissue expanders (STEs) has led to potentially improved utility and outcome [11]. STEs, first developed for use in human medicine in the 1970s and 1980s [23], function via fluid absorption by an osmotically active hydrogel core that generates a spontaneous volume increase by drawing in water from surrounding tissues. They do not require subcutaneous or external filling ports, thereby decreasing the risk of infection or additional handling and intervention. The rate of expansion depends on the composition of a silicone coating surrounding the hydrogel and is likely influenced by patient temperature and local fluid balance [25, 26]. Similarly, the extent of expansion is determined by the silicone coating, which acts to contain the expanding core [27]. The use of STEs is well established in human reconstructive surgery, and they have recently been used for malignant and benign tumor resection and primary wound closure in veterinary medicine [11, 25, 27, 28]. To the authors' knowledge, the use of STEs has not previously been reported in an exotic or nonmammal species.

## 2. Case Presentation

A 0.34 kg adult female Massasauga rattlesnake (*Sistrurus catenatus*) presented in August 2017 for evaluation of a ventral scale mass. The snake was wild-caught as a juvenile and had an estimated hatch date of 2004. After capture, it was kept at a local wildlife nature center for six years and then donated to the North Carolina Zoo in 2011, where it had been housed since that time. The snake's medical history included hypopyon in the left eye noted in 2012 that resolved with antibiotic therapy and mild conspecific trauma in 2015 that healed without intervention.

At the time of presentation, the animal was singly housed off-exhibit; caretakers reported normal demeanor and appetite. Physical exam while under manual restraint confirmed a semifirm, roughly ovoid mass that effaced a ventral scale to the left of midline 2/3 down the length of the body and extended below and elevated the adjacent scales. A crust overlaid the mass on the most severely affected scale. An ultrasound exam localized the mass to the subcutaneous tissues directly overlying the gallbladder, and subsequent evaluation of recorded images provided rough measurements (2.4 cm length × 1.2 cm width). For reference, the snake's ventral scale width and body height in the region of the mass measured 3.5 cm and 3.7 cm, respectively. The mass could be isolated from underlying internal organs and adjacent ribs using digital palpation. However, direct penetration into the coelomic cavity could not be ruled out based solely on palpation.

Anesthesia for biopsy and staging was performed approximately three weeks later. The cranial half of the snake was manually restrained via a plastic tube using standard safety protocols for the handling of venomous snakes at the North Carolina Zoo. It was then lightly anesthetized with alfaxalone (Alfaxan, 10 mg/mL, Jurox Inc., Kansas City, MO 64116, USA) 3 mg/kg IV. Whole body radiographs including orthogonal views revealed a cutaneous ovoid soft-tissue mass containing an irregular mineral opacity. Metastases were not observed. Ultrasonographic exam showed that the mass had grown since the previous exam and was now 3.2 × 1.8 × 1.2 cm. The remainder of the coelomic ultrasound revealed multiple ovarian follicles and no discernable metastases. Biopsies were performed to further characterize the mass. An intradermal ring block was administered around the mass using lidocaine (Lidocaine, 2%, Sparhawk Laboratories Inc., Lenexa, KS 66215, USA) 2.2 mg/kg diluted to 0.2 mL with saline. The snake received meloxicam (Loxicam, 5 mg/mL, Norbrook Inc., Overland Park, KS 66219, USA) 0.2 mg/kg IM. A 2 mm punch biopsy was used to produce two biopsy samples. Touch impressions of the biopsies were obtained prior to fixation in formalin. Cytology was consistent with a soft tissue sarcoma.

Blood was collected from the ventral tail vein and analyzed for complete blood count and plasma chemistry. Blood smear evaluation revealed a white cell estimate of 6970 cells/*μ*L with 15% heterophils, 39% lymphocytes, and 46% azurophils. Results were comparable to five routine samples previously collected from this individual between 2011 and 2015, aside from a slight increase in percent azurophils (46%, historic range 19-30%). This was attributed to chronic inflammation associated with the mass. Packed cell volume (PCV) was 44%, and platelets appeared adequate on the blood smear. Plasma chemistry was performed using a point-of-care analyzer (VetScan, Abaxis North America, Union City, CA 94587, USA) and showed a hypercalcemia of >4 mmol/L (>16 mg/dL) that was consistent with active follicle production. Creatine kinase (CK) was also elevated at 1151 U/L. The CK elevation may have resulted from anesthetic injections or local muscle disruption due to the mass. The snake recovered routinely from the procedure and was returned to its normal enclosure. The biopsy sites appeared to be healing appropriately at recheck nine days later. Initial biopsy results reported an ulcerated spindle cell sarcoma with myxomatous stroma and a small amount of osteoid and mineralized material suggestive of osteosarcoma. No mitotic figures were noted, but neoplastic cells were observed infiltrating into the adjacent stroma.

**Figure 1:**
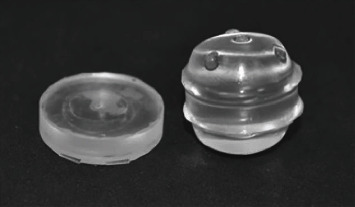
Self-inflating hygroscopic tissue expander (Oxtex Ltd.) before and after expansion.

Based on the diagnosis of a sarcoma with no concurrent evidence of metastases and apparently good systemic health, surgical excision was planned. Currently, there are no studies critically evaluating the typical biological behavior, management strategies, or long-term outcomes for the treatment of sarcomas in reptiles [29]. Without specific recommendations, a surgical plan was developed based on the current small animal guidelines [30–33]. The plan included excision with 1-2 cm lateral margins and the removal of the coelomic membrane directly below the mass, with the possibility for postoperative radiation or chemotherapy if clean margins were not obtained. To achieve these margins in the craniocaudal plane, 2-3 ventral scales would need to be removed both cranially and caudally to the mass in addition to the scales from which the mass arose. It was determined that excess tension across the excision site would likely preclude primary closure of the defect based on palpation of the area during surgical planning. Further, excess tension may have significantly impaired postoperative healing and mobility. These concerns, along with the goal of providing ample dermal coverage to facilitate primary closure of the excision site, necessitated the use of skin stretching or expansion techniques of the lateral scales adjacent to the mass. A hygroscopic self-inflating tissue expander (Expaniderm, Oxtex Limited, Witney OX29 7DX, UK) (Figure 1) was chosen in this case to provide controlled skin expansion without necessitating additional interventions such as bandaging or insufflation-port maintenance. This STE was recently developed for use in domestic mammals. It is designed with a thin silicone coating overlying a hydrogel core that absorbs fluid from surrounding tissues via osmosis, resulting in spontaneous expansion. The STE used in this report follows a relatively predictable rate of expansion in mammals: expansion begins at two to four days after a postoperative delay period, followed by a period of linear growth, achieving maximal expansion between two and four weeks.

**Figure 2:**
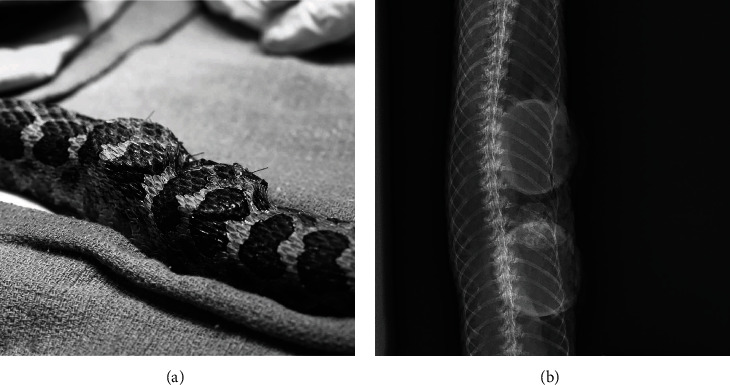
Immediate postoperative (a) photograph and (b) dorsoventral radiograph of a Massasauga rattlesnake (*Sistrurus catenatus*) with two self-inflating hygroscopic tissue expanders implanted subcutaneously under the lateral scales.

Implant placement was performed eight weeks after initial presentation. Anesthesia was induced with alfaxalone 5 mg/kg IV in the ventral tail vein, and anesthesia was maintained via isoflurane 0.25-1.0%. Two additional IV boluses of alfaxalone, 2.7 mg/kg each, and one dose of ketamine (KetaVed, 100 mg/mL, Vedco Inc., Saint Joseph, MO 64507, USA) 5.5 mg/kg IV were needed to maintain a surgical plane of anesthesia. Apnea developed and the snake was intubated with a 2.1 mm endotracheal tube to provide intermittent positive pressure ventilation. Lidocaine was administered subcutaneously at the incision site for local anesthesia. Additional treatments included meloxicam 0.3 mg/kg IM, lactated ringer's solution (LRS) 22 mL/kg SQ, and butorphanol (Butorphanol tartrate, 10 mg/mL, Bayer Corporation, Whippany, NJ 07981, USA) 1 mg/kg IM. Butorphanol was initially chosen by the primary clinician as part of a multimodal analgesic plan. This opioid is reported to provide pain control in snakes only at high doses [34] and is therefore unlikely to have contributed significantly to analgesia in this case. Ceftiofur crystalline free acid (Excede, 100 mg/mL, Zoetis Animal Health LLC, Parsippany, NJ 07054, USA) 14 mg/kg IM was given for perioperative antibiotics. A nonsterile dummy version of the implant provided by the manufacturer was used to outline placement locations adjacent to the mass over the left body wall. Strict aseptic technique was utilized to prevent bacterial contamination of the implant site. A 2 cm skin incision was made using a 15-scalpel blade between the second and third rows of scales on the left lateral body wall adjacent and slightly caudal to the mass. A subcutaneous pocket was created dorsal to the incision using blunt dissection, taking caution not to disrupt the local blood supply. The pocket was just large enough to accommodate the implant without causing excessive tension at the skin closure site. There was a mild-moderate amount of hemorrhage from the subcutaneous vessels. Hemostasis was achieved with a combination of cautery and direct pressure. The sterile STE implant measuring 27 mm diameter × 5 mm height was placed into the pocket directly over visible trunk musculature, allowing a 0.25 mm gap between the edge of the implant and incision site per manufacturer recommendations. The area was flushed with sterile saline, and the skin was then closed in a single layer using 4-0 polydioxanone suture (PDS II, Ethicon Inc., Johnson and Johnson Gateway LLC, Piscataway, NJ 08854, USA) in an interrupted horizontal mattress pattern. A second implant was placed approximately 1 cm cranial to the first using the same surgical technique (Figure 2(a)). Postoperative radiographs were taken to provide baseline images showing placement of the semiradiopaque implants (Figure 2(b)). Postoperative PCV and total solids were 28% and 5.8 g/dL, respectively. This drop in PCV as compared to four weeks prior was discordant with the observed intraoperative hemorrhage. It was suspected that anemia of chronic disease or paraneoplastic disease may have contributed to this mild anemia [35]. Recovery was prolonged, but smooth. The snake received meloxicam 0.3 mg/kg IM for one day postoperatively. The implants were monitored daily by keeper staff and examined one to two times weekly by a veterinarian throughout the expansion period.

**Figure 3:**
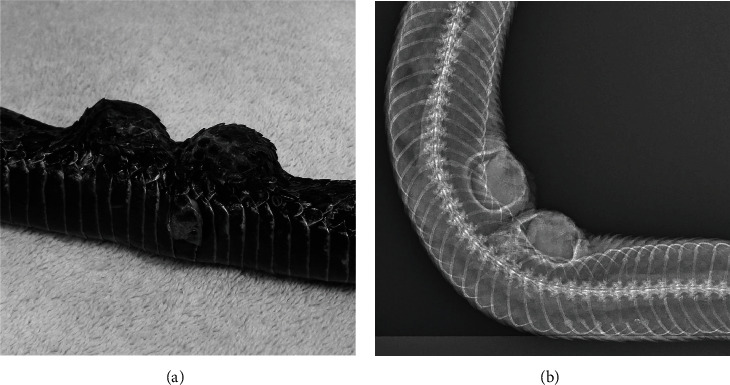
(a) Photograph and (b) dorsoventral radiograph of a Massasauga rattlesnake (*Sistrurus catenatus*) showing maximal expansion of two self-inflating hygroscopic tissue expanders four weeks after implant placement.

In mammals, the implants begin to expand 2-4 days after placement and then show a constant rate of expansion up to 14 days. However, at room temperature, expansion does not begin until 14 days and is complete at 28 days [36]. Though the snake was provided with a high-temperature basking area, the average temperature in the enclosure was 28°C (82°F). Based on consultation with the manufacturer, SQ fluids (LRS, 9 mL/kg) were administered adjacent to the implants on day seven, 14, and 23 postoperatively to promote fluid uptake and expansion. A maximal expansion of 16 mm in height was achieved in 4 weeks (Figure 3(a)), and radiographs showed minimal displacement of the underlying ribs (Figure 3(b)). The snake shed without difficulty 28 days after implant placement. During the tissue expansion period, the animal reportedly ate all meals offered and did not exhibit any changes in demeanor.

**Figure 4:**
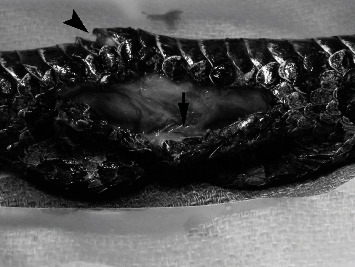
Massasauga rattlesnake (*Sistrurus catenatus*). Intraoperative photograph taken after extraction of two self-inflating hygroscopic tissue expanders. The excess expanded skin is visible on the near side of the incision. There is a thin band of soft tissue between the two implant sites (arrow). The ventral surface of the neoplasm is displacing the ventral scales (arrowhead).

**Figure 5:**
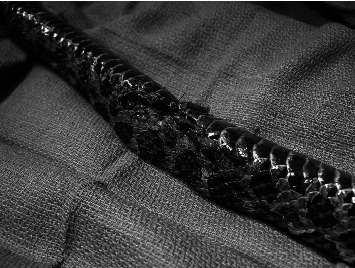
Postoperative photograph showing incorporation of expanded lateral skin into an osteosarcoma excision site in a Massasauga rattlesnake (*Sistrurus catenatus*).

**Figure 6:**
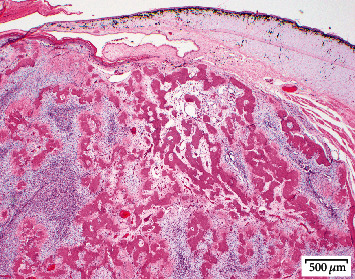
Massasauga rattlesnake (*Sistrurus catenatus*). Low-magnification (20x) photomicrograph of a dermal osteosarcoma composed of a well-demarcated, expansile mass of haphazardly arranged spindle cells interspersed between irregular islands of bone and myxomatous matrix.

Implant removal and tumor excision surgery was performed 36 days after implant placement. Anesthesia was induced and maintained as previously outlined. To access the implants, the skin was incised along the previous incision line. A combination of blunt and sharp dissection was used to disrupt a thin tissue membrane that had formed surrounding the implants. The implants were easily removed with gentle traction. The associated subcutaneous tissues and skin appeared vital with no overt fibrosis of the expanded skin and surrounding tissues (Figure 4). When laid over the region of the mass, the leading edge of the expanded skin reached 1-1.5 cm across the ventral scales. The skin maintained what appeared to be near-normal pliability. The skin could be stretched greater than 50% of the ventrum's width if gentle tension was applied to the leading edge. The skin margin incorporated into the previously healed incision site was sharply excised, and the incision was extended cranially and caudally by approximately 0.5 cm. The underlying coelomic membrane was then identified and incised. The internal surface of the mass was visualized and grossly appeared to extend to, but not through, the coelomic membrane. Brief exploration of the visible coelomic cavity revealed normal distal hepatic parenchyma, gall bladder, coelomic fat, and associated connective tissues. The ventral abdominal vein ran directly through the mass and was therefore ligated cranially and caudally with a combination of hemoclips (Weck Hemoclip System, Rica Surgical Products Inc., Schiller Park, IL 60176) and encircling sutures of 4-0 PDS. The mass was removed with a minimum of 1 cm circumferential margins. The underlying coelomic membrane, which was adhered to the mass, was also surgically excised. Cautery was used to manage moderate intraoperative hemorrhage. The skin was opposed in a Y-shape using 4-0 PDS with a horizontal mattress pattern, incorporating the expanded lateral skin into the center of the closure to decrease tension across the incision site (Figure 5). The skin was opposed easily with minimal tension. Tissue glue was applied sparingly to the cranial and caudal-most aspects of the incision. Based on the compliance and extent of the remaining expanded skin, it was noted that primary closure would still have been possible with significantly wider excisional margins (1.5-2 cm). However, due to the duration of anesthesia, observed intraoperative hemorrhage, and perceived adequate margins, further excision was not pursued. Additional treatments included meloxicam 0.3 mg/kg IM, ceftiofur crystalline free acid 15 mg/kg IM, and LRS 10 mL/kg IV and 30 mL/kg SQ. Recovery was smooth. Histologic examination of the mass confirmed a diagnosis of osteosarcoma (Figure 6). There was hyperkeratosis of the overlying epidermis with occasional large colonies of cocci bacteria. Narrow margins were observed along the cranial, caudal, and lateral margins and neoplastic cells extended to the edge of the deep surgical margin.

Postoperative care included meloxicam 0.3 mg/kg IM and LRS 20 mL/kg SQ daily for four doses. The remaining loose skin at the implant sites over the left lateral body wall was notably decreased by two days postoperatively. The surgical site appeared to be healing well at multiple veterinary checks during the next two weeks, and the snake's demeanor remained bright. However, it was found dead by keepers 14 days postoperatively. The animal had refused a meal several days prior, but otherwise had not shown clear premonitory symptoms.

Gross postmortem examination revealed a mild amount of translucent coelomic fluid and adequate internal fat stores. The lungs and kidney contained numerous pinpoint to 0.02 cm diameter, discrete white masses, and a larger mass was identified within the cardiac ventricular lumen measuring 0.03 × 0.05 cm. The liver was irregular with multifocal areas of parenchymal collapse. The surgical site contained some bruising but otherwise appeared appropriate for this stage of healing.

Metastatic neoplasia was suspected based on gross postmortem exam. However, histology revealed that the intracardiac heart mass corresponded to an area of severe, fibrinous endocarditis with mixed bacteria (vegetative endocarditis). The masses within the liver and lungs, as well as smaller foci in the kidneys, were revealed to be foci of necrosis and heterophilic inflammation with intralesional mixed bacteria. These foci were frequently associated with intravascular fibrin thrombi consistent with embolic sepsis. Histology of the surgical closure site showed marked inflammation and numerous reactive changes, but no overt neoplastic cells or bacteria were identified. The precise cause of the bacterial endocarditis with systemic emboli is unknown. Infection may have entered via the biopsy sites, implant or mass excision sites, or via the ulceration on the surface of the tumor itself. Alternatively, infection could have resulted from an entirely unrelated previous subclinical disease. Immunosuppression induced by the tumor, implant placement, or the stress of handling may have increased susceptibility to infection.

The postoperative mortality provided an opportunity for histologic examination of the expanded skin two weeks after implant removal. The expanded donor site contained mild, subacute hemorrhage and edema with minimal inflammatory infiltrates.

## 3. Discussion

To the authors' knowledge, this is the first description of tissue expansion for preoperative surgical planning in a reptile and the first clinical use of STEs in a nondomestic animal.

In the case reported here, the expansion period was slightly protracted, as might be expected for a reptile. In contrast to *in vivo* use in mammals, the manufacturer reports that implants held at room temperature typically begin expanding at 2 weeks [36]. Therefore, an ambient temperature of 28°C (82°F) in the snake's enclosure likely influenced the expansion rate. The administration of SQ fluids adjacent to the implants appeared to enhance expansion, suggesting that fluid balance also played a role. The benefits of a slow and controlled expansion as was seen in this case are the decreased risks of dermal necrosis, dehiscence, extrusion, and discomfort from acute stretch.

The manufacturer recommends that the implants are left in for two weeks in preparation for surgery, though both shorter (13 days) and longer periods (42 days) have been reported in dogs [11]. Complications of skin expansion in domestic animals include fibrous capsule formation, local infection, necrosis of overlying skin due to compression of the local blood supply, dehiscence and subsequent implant extrusion, compression of underlying bone or soft tissues, and evidence of discomfort [11, 15, 36]. In the case presented here, STEs placed subcutaneously on the lateral aspect of the body wall provided ample skin expansion in 4 weeks with no overt complications. The overlying skin appeared healthy, implant migration did not occur, and normal healing of the incision site was observed. There was no grossly visible or histologic evidence of fibrosis, nor devitalized or infected tissue observed when the implants were removed. Radiographs showed minimal displacement of underlying ribs. Additionally, the snake maintained normal appetite and behavior during the expansion period. At the time of tumor resection 5 weeks after implant placement, the expanded skin appeared to maintain a near-normal degree of compliance. The amount of excess skin would have allowed for resurfacing of an even larger defect in this case.

The success of tissue expansion via STEs in this patient is attributed in part to the innate compliance of ophidian skin, which has evolved to accommodate locomotion, macrophagy, and egg production. The mechanical properties of snake skin vary widely between locations on the body and among species [37, 38]. For example, in garter snakes, the skin cranial to the pylorus exhibits increased stretch compared to the skin caudally on the body [38]. Therefore, the success of this skin expansion technique may not extrapolate to all snake species or anatomic locations. Consideration should also be made for potential discomfort induced by implant placement and expansion. The slow, progressive expansion of STEs limits acute strain on the tissues and is reportedly nonpainful in humans [28, 39–41], though it is assumed that some degree of discomfort occurs with acute stretch and surgical trauma during initial placement. Another drawback of this reconstructive technique is the necessity for two general anesthetic and surgical procedures, one for implant placement and one for the definitive reconstruction. This increases the risk of perioperative complications and may allow for tumor progression or metastasis during the delay period in the case of neoplastic disease.

Though this individual died of sepsis seven weeks after implant placement, the implants were considered an unlikely source of direct infection based on the healthy appearance of the implant site at removal, lack of clinical signs during the expansion period, and lack of inflammation in this area at time of death. Additionally, strict aseptic technique and perioperative antibiotics were administered. It is suspected that the tumor surface, biopsy sites, or excision sites represent the most likely sources of bacterial entry and subsequent endocarditis. The authors feel that STEs have strong potential as a viable tool for resurfacing surgical defects in snakes despite the two-week postoperative mortality in this case.

Finally, this case represents the first report of presumed-extraskeletal osteosarcoma in a crotalid. Primary skeletal neoplasms such as fibrosarcoma and chondrosarcoma are prevalent in snakes [42, 43]. However, osteosarcomas were diagnosed in only three of 325 snakes with neoplasia submitted to a specialty diagnostic service and are infrequently reported in the literature [42, 44–46]. Osteosarcomas in snakes typically arise from vertebrae and ribs, rarely from extraskeletal sites [42, 44, 47]. In this clinical report, the osteosarcoma increased in size by roughly 33% from the time of presentation to staging four weeks later in this clinical report. However, measurements of the excised mass confirmed that minimal additional increase occurred thereafter. Although the tumor appeared locally invasive, the mitotic rate was low, and no metastases were evident on antemortem and postmortem examinations. A thorough review of ophidian osteosarcomas will be necessary to describe the typical biological behavior of this tumor and better inform treatment decisions.

This paper highlights the utility of STEs for the excision of a locally invasive tumor in a rattlesnake. Specifically, preoperative tissue expansion permitted wide surgical margins and primary closure of the resulting defect. The side effects of STEs reported in domestic animals were minimal or not observed in this case. It is possible that stress and immunosuppression prompted by implant placement and postoperative care may have increased this animal's susceptibility to infection. Therefore, prognosis, feasibility of implant placement, patient demeanor, and immune status should be considered when choosing reptilian candidates for this novel technique.

## Data Availability

All relevant data are presented in the case description.
